# Does selective intraoperative music reduce pain following abdominal wall reconstruction? A double-blind randomized controlled trial

**DOI:** 10.1007/s10029-024-03092-y

**Published:** 2024-06-18

**Authors:** Sara M. Maskal, Corey K. Gentle, Ryan C. Ellis, Chao Tu, Michael J. Rosen, Clayton C. Petro, Benjamin T. Miller, Lucas R. A. Beffa, Jenny H. Chang, Nir Messer, Megan Melland-Smith, Johannes Jeekel, Ajita S. Prabhu

**Affiliations:** 1 Cleveland Clinic Foundation, Crile Building, A100, 2049 E 100th Street, Cleveland, OH 44195 USA; 2 Erasmus Medical Center, Erasmus University, Rotterdam, Netherlands

**Keywords:** Music, Postoperative pain, Abdominal wall reconstruction, Multimodal analgesia, Opioid consumption, Ventral hernia repair

## Abstract

**Purpose:**

Although intraoperative music is purported to mitigate postoperative pain after some procedures, its application has never been explored in abdominal wall reconstruction (AWR). We sought to determine whether intraoperative music would decrease early postoperative pain following AWR.

**Methods:**

We conducted a placebo-controlled, patient-, surgeon-, and assessor-blinded, randomized controlled trial at a single center between June 2022 and July 2023 including 321 adult patients undergoing open AWR with retromuscular mesh. Patients received noise-canceling headphones and were randomized 1:1 to patient-selected music or silence after induction, stratified by preoperative chronic opioid use. All patients received multimodal pain control. The primary outcome was pain (NRS-11) at 24 ± 3 h. The primary outcome was analyzed by linear regression with pre-specified covariates (chronic opioid use, hernia width, operative time, myofascial release, anxiety disorder diagnosis, and preoperative STAI-6 score).

**Results:**

178 patients were randomized to music, 164 of which were analyzed. 177 were randomized to silence, 157 of which were analyzed. At 24 ± 3 h postoperatively, there was no difference in the primary outcome of NRS-11 scores (5.18 ± 2.62 vs 5.27 ± 2.46, p = 0.75). After adjusting for prespecified covariates, the difference of NRS-11 scores at 24 ± 3 h between the music and silence groups remained insignificant (p = 0.83). There was no difference in NRS-11 or STAI-6 scores at 48 ± 3 and 72 ± 3 h, intraoperative sedation, or postoperative narcotic usage.

**Conclusion:**

For patients undergoing AWR, there was no benefit of intraoperative music over routine multimodal pain control for early postoperative pain reduction.

**Trial registration:**

ClinicalTrials.gov: NCT05374096.

**Supplementary Information:**

The online version contains supplementary material available at 10.1007/s10029-024-03092-y.

## Introduction

Open abdominal wall reconstruction (AWR) with retromuscular mesh placement is notoriously painful, and the postoperative course often involves high rates of opioid consumption in both inpatient and outpatient recovery [1, 2]. Although multimodal pain regimens and judicious use of opioid medications have remained a priority and the mainstay of care, the persistent presence of opioids postoperative pain management highlights the need for alternative, lower-risk interventions. Music has garnered attention as one such intervention for mitigating postoperative pain.

Perioperative music has demonstrated multiple benefits including reductions in pain, anxiety, and opioid consumption [3–6]. Two randomized trials involving inguinal hernia repairs showed reductions in patient-reported pain and analgesia consumption with perioperative music therapy [7, 8]. Patient-reported anxiety has also shown improvement with music exposure prior to general anesthesia [9] and patients listening to music postoperatively after open hernia repair in an ambulatory surgery center demonstrated lower cortisol levels compared to controls [10]. While the benefits of music for relaxation may seem apparent for conscious patients, there is evidence that even under deep sedation, the auditory cortex has demonstrated reactivity to auditory stimuli [11, 12]. In a meta-analysis of randomized controlled trials evaluating intraoperative auditory stimuli from multiple surgical disciplines, Fu et al. reported that intraoperative music was associated with reduced postoperative pain and opioid requirements [13]. However, the existing literature on perioperative music has not included patients undergoing AWR, an operation that involves significant dissection and retraction that is ostensibly painful. Thus, we designed a trial to investigate the effect of intraoperative music on pain after open AWR when compared to silence. We hypothesized that patient-selected music played exclusively intraoperatively would result in lower patient-reported pain at 24 h after surgery when compared with silence.

## Methods

### Study design and oversight

This was a registry-based, double-blinded, randomized controlled parallel group trial with a 1:1 allocation, where all enrolled patients received headphones and either patient-selected music or silence based on randomization. The Abdominal Core Health Quality Collaborative (ACHQC) and Research Electronic Data Capture (REDCap) database served as data collection platforms. All patients underwent open AWR with retromuscular mesh, performed by one of five coauthors (M.J.R., A.S.P., B.T.M., L.R.A.B., and C.C.P.) at a single tertiary academic center (Main Campus, Cleveland Clinic Foundation, Cleveland, Ohio). Institutional review board approval was granted prior to enrollment, and all study participants provided written informed consent. The study was registered on ClinicalTrials.gov (NCT05374096) and the trial was conducted and analyzed according to the Consolidated Standards of Reporting Trials guidelines for randomized controlled trials (RCT).

### Patients and study setting

All patients with ventral hernias requiring myofascial advancement flaps presenting to the surgeons’ clinics were screened for enrollment and were enrolled by treating physicians or qualified research personnel. Inclusion criteria were adult patients undergoing open retromuscular ventral, flank, or parastomal hernia repair with mesh, with myofascial release for a hernia width ≤ 20 cm. Exclusion criteria were primary language other than English, lack of English fluency, hearing impairment with or without use of hearing aids, neurologic condition precluding accurate assessment of postoperative pain and anxiety, and patients who were planned to remain intubated after surgery. The protocol was amended after trial commencement, prior to data analysis, to exclude patients who were randomized but did not undergo retromuscular AWR to account for cases where surgeons deferred definitive hernia repair for a staged approach at their discretion, which was anticipated to affect expected postoperative pain as the patients did not undergo open AWR as planned.

### Interventions

Patient-selected music and preoperative opioid use history in morphine milligram equivalents (MME) for the past month and year were documented preoperatively. On the day of surgery, general anesthesia was induced per the discretion of the anesthesiologist. Vasopressors, antihypertensives, chronotropic drugs, narcotics, and sedatives were given as clinically indicated. Noise-canceling headphones were placed on all patients by study coordinators after induction of anesthesia and prior to incision. Randomization was performed through RedCap by a study coordinator uninvolved in data collection or assessment, who then administered the appropriate treatment of patient-selected music or silence. To maintain blinding and minimize bias, the intervention was solely administered intraoperatively while patients were fully sedated. The same individual removed the headphones at the beginning of anesthesia emergence. The surgical team and anesthesia team were blinded to allocation and had no interaction with the headphones or music playing device. Intraoperative sedation and analgesia administered were per routine practices and were recorded. The anesthetist was asked if they believed there was music or silence administered through the headphones. All patients were given an intraoperative transversus abdominis plane block with plain bupivacaine by the operating surgeon and received postoperative multimodal analgesia according to our institutional Enhanced Recovery After Surgery pathway [14].

### Data collection

Patients remained blinded to their randomization postoperatively. A blinded assessor asked patients which treatment they thought they had received intraoperatively at 24 ± 3 h after surgery and administered Numeric Rating Scale (NRS-11) and State-Trait Anxiety Inventory (STAI-6) surveys at 24 ± 3 h, 48 ± 3 h, and 72 ± 3 h. NRS-11 is a validated scale from 0 to 10 with 0 corresponding to “no pain” and 10 corresponding to “the worst pain imaginable”, and various minimally important clinical differences have been previously reported in acute and chronic pain contexts ranging from 1.65 absolute point difference to 56% reductions [15–17]. STAI-6 is a six-item validated survey scaled from 20 to 80 with higher numbers indicating higher anxiety and a minimal clinically important difference (MCID) of 10 points [18, 19]. Patient analgesic requirements were recorded at 24 ± 3 h, 48 ± 3 h, and 72 ± 3 h. Data from the 30 ± 15 day postoperative visit were prospectively collected as standard of care, including: length of stay, ileus, bowel obstruction, pain requiring intervention, deep vein thrombosis/pulmonary embolism, stroke, sepsis, septic shock, myocardial infarction, cardiac arrest, urinary retention, urinary tract infection, renal insufficiency, acute renal failure, pneumonia, respiratory failure, ventilator > 48 h, surgical site infection (SSI), surgical site occurrence (SSO), surgical site occurrence requiring procedural intervention (SSOPI), reoperation, and readmission [20].

### Outcomes

The primary outcome was pain score, measured by NRS-11 score at 24 ± 3 h postoperatively. Secondary outcomes included NRS-11 scores at 48 ± 3, and 72 ± 3 h, anxiety measured using the STAI-6 questionnaire at 24 ± 3, 48 ± 3, and 72 ± 3 h. Cumulative pain in the first 24 h postoperatively was also measured using NRS-11 scores documented by nursing staff. Additional outcomes included opioid consumption in the first 72 h postoperatively, intraoperative sedative requirements, STAI-6 correlation with prespecified outcomes (postoperative NRS-11 pain scores, complication rates, length of stay, and readmission), and the relationship between patients guessing they had music intraoperatively and multiple outcomes (STAI-6 scores, NRS-11 pain scores, rate of complications, length of stay, and rates of readmission). An exploratory subgroup analysis was performed that only included patients without preoperative chronic opioid use or anxiety disorder to compare NRS-11 pain scores, STAI-6 scores, and opioid consumption in the first 72 h postoperatively based on treatment with music versus silence.

### Power calculation

Our study investigated the superiority of patient-selected music to reduce postoperative pain compared to no music. Based on a sample of 100 patients who underwent retromuscular AWR with myofascial release and mesh at our institution, the mean NRS-11 score was 4.52 ± 2.47 at 24 ± 12 h. Given the lack of a published MCID for NRS-11 in hernia surgery specifically, consensus opinion among the co-investigators was that a 20% reduction would be clinically meaningful. At 90% power while holding a type I error at 5%, a sample size of 320 total patients with 160 in each arm was required.

### Randomization and blinding

Randomization was generated by an institutional statistician (C.T.) and allocation was completed by a research coordinator (S.M.M., C.G., R.C.E., J.H.C., and N.M.). Eligible patients were randomized consecutively in a 1:1 fashion after confirming that headphones could be placed. Randomization was stratified based on chronic opioid use, defined as daily use for 90 days or more in the past year. A central concealed, random-block randomization scheme was housed in REDCap. A non-blinded research coordinator applied the allocated intervention. The surgical team, anesthesia team, postoperative assessor, and the patient remained blinded to randomization throughout the trial.

### Statistical analysis

Randomized groups were assessed for balance on baseline characteristics using the standardized differences, defined as the difference in means or proportions divided by the pooled standard deviation. An absolute standardized difference > 0.10 was considered imbalanced. Analysis was performed under the principles of intent to treat with no interim analyses planned.

The primary outcome of pain score on NRS-11 at 24 ± 3 h postoperatively was analyzed using linear regression with pre-specified covariates (chronic opioid use, baseline NRS-11 score, hernia width, operative time, myofascial release, pre-operative anxiety disorder diagnosis, and pre-operative STAI-6 anxiety score). The treatment effect of music versus silence on the STAI-6 anxiety questionnaire at 24 ± 3 h postoperatively was assessed using linear regression with pre-specified covariates (chronic opioid use, hernia width, operative time, myofascial release, pre-operative anxiety disorder diagnosis, and pre-operative STAI-6 anxiety score). Cumulative pain during the first 24 h post operatively was analyzed as the area under the curve using Sum Pain Intensity Differences Area Calculation. The treatment effect of intraoperative music on STAI-6 scores over the first 72 ± 3 h was analyzed using a mixed effect linear regression model with treatment group and pre-specified covariates as fixed effect and the repeated measurements as random effect. The remaining outcomes were analyzed with linear and logistic regressions. All analyses were performed using R version 4.3.1 (The R Foundation). P < 0.05 was considered significant.

## Results

### Patients

**Fig. 1:**
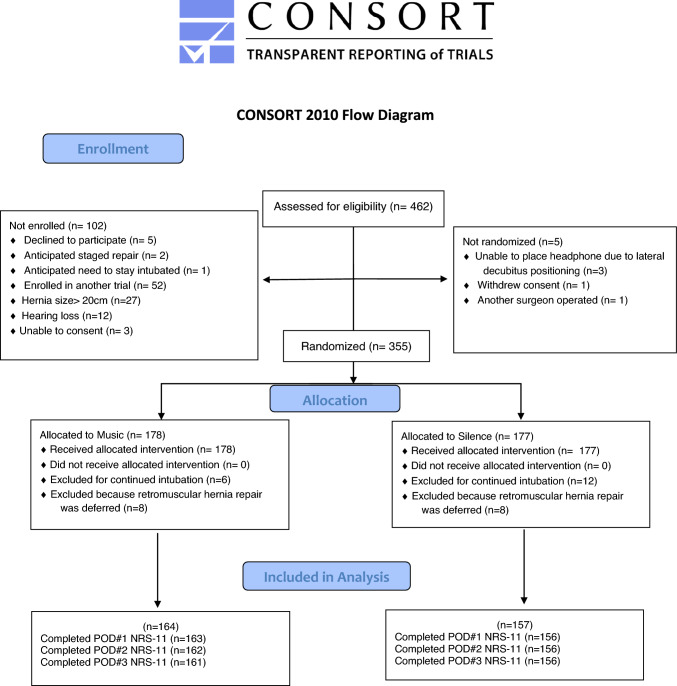
Consolidated Standards of Reporting Trials Diagram

Enrollment commenced in June 2022 and ended in July 2023 due to trial completion. Of 355 patients enrolled, 34 patients were excluded after randomization, 14 in the music arm and 20 in the silence arm. A total of 164 patients in the music arm and 157 patients in the silence arm were included in the analysis (Fig. 1). All randomized patients received the intended treatment. Patient demographics and operative details were well balanced (Table 1). The mean age was 59.7(± 11.9) years, mean BMI was 33.0(± 6.21) kg/m^2^, and 136(42.4%) participants were male. Mean hernia width was 14.5(± 4.60) cm, and 50(15.6%) patients underwent a parastomal hernia repair. Mean operative time was 3.87(± 1.33) hours. There was no significant difference in postoperative outcomes between groups (Supplement 1).

**Table 1:** Patient demographics and operative characteristics

	[ALL]	Music	Silence
	*N* = *321*	*N* = *164*	*N* = *157*
Age (years), mean (SD)	59.7 (11.9)	59.9 (11.4)	59.6 (12.3)
Sex, n (%)			
Male	136 (42.4%)	71 (43.3%)	65 (41.4%)
Female	185 (57.6%)	93 (56.7%)	92 (58.6%)
Race, n (%)			
Asian or Pacific Islander	1 (0.32%)	0 (0.00%)	1 (0.65%)
Black	15 (4.81%)	5 (3.16%)	10 (6.49%)
Hispanic	6 (1.92%)	3 (1.90%)	3 (1.95%)
White	289 (92.6%)	149 (94.3%)	140 (90.9%)
Middle Eastern	1 (0.32%)	1 (0.63%)	0
Preoperative chronic opioid use, n (%)	50 (15.6%)	27 (16.5%)	23 (14.6%)
Opioid use (past month) in MME, mean (SD)	750 (9593)	318 (1409)	1201 (13,649)
Opioid use (past year) in MME, mean (SD)	9115 (115,122)	4035 (17,092)	14,422 (163,784)
Anxiety disorder, n (%)	96 (29.9%)	49 (29.9%)	47 (29.9%)
Hypertension, n (%)	212 (66.0%)	102 (62.2%)	110 (70.1%)
Diabetes, n (%)	85 (26.5%)	38 (23.2%)	47 (29.9%)
Chronic Obstructive Pulmonary Disease, n (%)	30 (9.35%)	11 (6.71%)	19 (12.1%)
Functional status, n (%)			
Independent	314 (97.8%)	162 (98.8%)	152 (96.8%)
Partially Dependent	6 (1.87%)	2 (1.22%)	4 (2.55%)
Totally Dependent	1 (0.31%)	0 (0.00%)	1 (0.64%)
Antiplatelet use, n (%)	22 (6.85%)	7 (4.27%)	15 (9.55%)
Anticoagulant use, n (%)	30 (9.35%)	11 (6.71%)	19 (12.1%)
Steroid use, n (%)	23 (7.17%)	12 (7.32%)	11 (7.01%)
Nicotine use, n (%)	37 (11.5%)	17 (10.4%)	20 (12.7%)
Inflammatory bowel disease, n (%)	35 (10.9%)	18 (11.0%)	17 (10.8%)
Recurrent hernia, n (%)	186 (57.9%)	97 (59.1%)	89 (56.7%)
Number of previous hernia repairs, mean (SD)	2.23 (1.15)	2.23 (1.07)	2.24 (1.24)
Previous component separation, n (%)	45 (14.0%)	23 (14.0%)	22 (14.0%)
ASA Class*, n (%)			
2	47 (14.6%)	28 (17.1%)	19 (12.1%)
3	268 (83.5%)	135 (82.3%)	133 (84.7%)
4	6 (1.87%)	1 (0.61%)	5 (3.18%)
Wound Status, n (%)			
Clean	227 (70.7%)	118 (72.0%)	109 (69.4%)
Clean-contaminated	58 (18.1%)	26 (15.9%)	32 (20.4%)
Contaminated	35 (10.9%)	19 (11.6%)	16 (10.2%)
Dirty/Infected	1 (0.31%)	1 (0.61%)	0 (0.00%)
Concomitant procedure, n (%)	65 (20.2%)	34 (20.7%)	31 (19.7%)
Operating time (hours), mean (SD)	3.69 (1.02)	25 (15.2%)	24 (15.3%)
Intraoperative complication, n (%)	18 (5.61%)	7 (4.27%)	11 (7.01%)
Hernia length(cm), mean (SD)	21.8 (6.28)	28 (17.1%)	19 (12.1%)
Hernia width (cm), mean (SD)	14.5 (4.60)	135 (82.3%)	133 (84.7%)
Stoma present, n (%)	57 (17.8%)	30 (18.3%)	27 (17.2%)
Transversus abdominis release, n (%)	319 (99.4%)	163 (99.4%)	156 (99.4%)
Mesh length (cm), mean (SD)	34.1 (7.37)	34.0 (7.54)	34.3 (7.22)
Mesh width (cm), mean (SD)	34.4 (8.12)	34.2 (8.17)	34.6 (8.09)
Transversus abdominis release laterality, n (%)			
Bilateral	300 (95.2%)	153 (96.2%)	147 (94.2%)
Unilateral	15 (4.76%)	6 (3.77%)	9 (5.77%)
Posterior rectus sheath incision alone, n (%)	6 (1.87%)	5 (3.05%)	1 (0.64%)
Transfascial fixation, n (%)	49 (15.3%)	25 (15.2%)	24 (15.3%)
Fascial closure, n (%)	309 (96.3%)	157 (95.7%)	152 (96.8%)
Incisional hernia, n (%)	315 (98.1%)	161 (98.2%)	154 (98.1%)
Parastomal hernia, n (%)	50 (15.6%)	28 (17.1%)	22 (14.0%)
Lateral hernia only, n (%)	14 (4.4%)	8 (4.9%)	6 (3.8%)
Incisional and parastomal hernia, n (%)	45 (14.0%)	25 (15.2%)	20 (12.7%)

*ASA American Society of Anesthesiologists

### Primary outcome

**Fig. 2:**
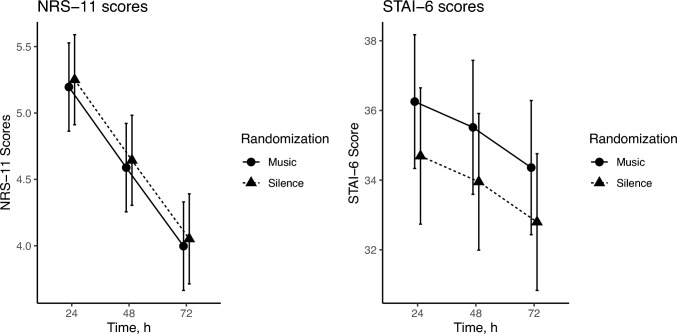
Pain (NRS-11) and anxiety (STAI-6) scores over the first 72 h postoperatively

At 24 ± 3 h postoperatively, there was no difference in the primary outcome of NRS-11 pain scores (5.18 ± 2.62 vs 5.27 ± 2.46, p = 0.75). After adjusting for prespecified covariates in a linear regression model, the difference of NRS-11 scores at 24 ± 3 h between the music and silence groups remained statistically insignificant (p = 0.83) (Fig. 2).

### Secondary outcomes

*Anxiety and pain:* STAI-6 scores at 24 ± 3 h postoperatively showed no difference between the music and silence groups (36.4 ± 15.0 vs 34.5 ± 13.7, p = 0.25), which was confirmed using a linear regression model adjusting for prespecified covariates (p = 0.39). In the mixed effect linear regression model, there was no difference in STAI-6 scores over the first 72 ± 3 h between the music and silence groups (p = 0.21) (Fig. 2). In the mixed effect linear regression model, there was no difference in NRS-11 scores over the first 72 ± 3 h between the music and silence groups (p = 0.80) or in the cumulative pain score in the first 24 h (Fig. 2).

*Additional secondary outcomes:* Results related to anesthesia requirements and assessments, analgesia requirements, and correlations of patient anxiety and allocation guess with outcomes are summarized in Tables 2, 3, 4, 5.

**Table 2:** Intraoperative sedation and analgesia

	[ALL]	Music	Silence	p-value
	*N* = *321*	*N* = *164*	*N* = *157*	
Intraoperative propofol (mg), mean (SD)	296 (311)	280 (229)	313 (378)	0.348
Intraoperative etomidate(mg), mean (SD)	0.47 (3.00)	0.23 (2.09)	0.73 (3.71)	0.145
Intraoperative precedex(mg), mean (SD)	0.82 (10.3)	1.60 (14.4)	0.00 (0.00)	0.158
Intraoperative MAC, mean (SD)	0.76 (0.13)	0.76 (0.12)	0.75 (0.15)	0.570
Intraoperative benzodiazepine(mg), mean (SD)	0.99 (0.98)	0.94 (0.98)	1.04 (0.99)	0.368
Intraoperative opioid (MME), mean (SD)	74.9 (46.2)	77.1 (59.2)	72.7 (26.5)	0.392
Anesthesia guess, n (%)				0.568
Music	180 (56.4%)	95 (58.3%)	85 (54.5%)	
Silence	139 (43.6%)	68 (41.7%)	71 (45.5%)

**Table 3:** Postoperative surveys and analgesia

	[ALL]	Music	Silence	p-value
	*N* = *321*	*N* = *164*	*N* = *157*	
Preoperative NRS-11, mean (SD)	2.93 (2.76)	2.77 (2.70)	3.11 (2.82)	0.271
Preoperative STAI-6, mean (SD)	40.3 (14.1)	41.2 (13.9)	39.4 (14.4)	0.254
NRS-11 at 24 ± 3 h, mean (SD)	5.22 (2.54)	5.18 (2.62)	5.27 (2.46)	0.748
STAI-6 at 24 ± 3 h, mean (SD)	35.5 (14.4)	36.4 (15.0)	34.5 (13.7)	0.247
Patient allocation guess, n (%)				0.308
Music	135 (42.6%)	64 (39.5%)	71 (45.8%)	
Silence	182 (57.4%)	98 (60.5%)	84 (54.2%)	
Opioid consumption (MME) at 24 h, mean (SD)	181 (289)	202 (384)	159 (128)	0.172
Tylenol consumption (mg) at 24 h, mean (SD)	2802 (1090)	2870 (983)	2731 (1191)	0.255
NSAID consumption (mg) at 24 h, mean (SD)	26.6 (180)	27.3 (204)	25.9 (152)	0.943
Gabapentin consumption (mg) at 24 h, mean (SD)	432 (314)	466 (350)	397 (268)	0.047
NRS-11 at 48 ± 3 h, mean (SD)	4.61 (2.56)	4.42 (2.64)	4.81 (2.47)	0.177
STAI-6 at 48 ± 3 h, mean (SD)	34.6 (14.7)	35.2 (15.0)	34.0 (14.5)	0.464
Opioid consumption (MME) at 48 h, mean (SD)	282 (435)	312 (572)	252 (213)	0.213
Tylenol consumption (mg) at 48 h, mean (SD)	5530 (1759)	5636 (1546)	5420 (1955)	0.274
NSAID consumption(mg) at 48 h, mean (SD)	57.6 (366)	64.8 (432)	50.0 (283)	0.716
Gabapentin consumption (mg) at 48 h, mean (SD)	1118 (650)	1191 (741)	1042 (531)	0.039
NRS-11 at 72 ± 3 h, mean (SD)	4.01 (2.58)	3.95 (2.54)	4.08 (2.63)	0.663
STAI-6 at 72 ± 3 h, mean (SD)	33.4 (14.8)	35.1 (15.1)	31.7 (14.3)	0.037
Opioid consumption (MME) at 72 h, mean (SD)	409 (1161)	381 (620)	438 (1537)	0.666
Tylenol consumption(mg) at 72 h, mean (SD)	8255 (2600)	8368 (2330)	8137 (2858)	0.431
NSAID consumption(mg) at 72 h, mean (SD)	97.9 (505)	90.1 (464)	106 (546)	0.779
Gabapentin consumption (mg) at 72 h, mean (SD)	1742 (993)	1840 (1112)	1639 (842)	0.068
Received postoperative cyclobenzaprine, n (%)	46 (14.3%)	23 (14.0%)	23 (14.6%)	1.000
Cyclobenzaprine consumption (mg) at 24 h, mean (SD)	7.93 (8.54)	8.91 (9.29)	6.96 (7.80)	0.443
Cyclobenzaprine consumption(mg) at 48 h, mean (SD)	18.9 (14.8)	19.8 (15.8)	18.0 (14.0)	0.695
Cyclobenzaprine consumption(mg) at 72 h, mean (SD)	31.0 (20.6)	31.5 (22.3)	30.4 (19.3)	0.861
Received postoperative methocarbamol, n (%)	73 (22.7%)	36 (22.0%)	37 (23.6%)	0.832
Methocarbamol consumption (mg) at 24 h, mean (SD)	630 (697)	653 (745)	608 (658)	0.787
Methocarbamol consumption (mg) at 48 h, mean (SD)	1682 (1411)	1764 (1464)	1601 (1374)	0.626
Methocarbamol consumption(mg) at 72 h, mean (SD)	2764 (2010)	2701 (2019)	2824 (2027)	0.796
Received postoperative benzodiazepine, n (%)	33 (10.3%)	19 (11.6%)	14 (8.92%)	0.547
Benzodiazepine consumption(mg) at 24 h, mean (SD)	1.58 (3.54)	1.00 (1.15)	2.36 (5.27)	0.360
Benzodiazepine consumption(mg) at 48 h, mean (SD)	3.79 (6.91)	2.37 (2.29)	5.71 (10.2)	0.247
Benzodiazepine consumption(mg) at 72 h, mean (SD)	5.58 (10.8)	3.21 (2.42)	8.79 (16.1)	0.220
Received postoperative lidocaine patch, n (%)	72 (22.4%)	33 (20.1%)	39 (24.8%)	0.379
Number of lidocaine patches at 24 h, mean (SD)	0.50 (0.50)	0.52 (0.51)	0.49 (0.51)	0.816
Number of lidocaine patches at 48 h, mean (SD)	1.24 (0.78)	1.18 (0.81)	1.28 (0.76)	0.592
Number of lidocaine patches at 72 h, mean (SD)	2.14 (0.84)	2.09 (0.84)	2.18 (0.85)	0.660
Received postoperative ketamine, n (%)	20 (6.23%)	14 (8.54%)	6 (3.82%)	0.130
Ketamine consumption (mg) at 24 h, mean (SD)	206 (260)	163 (198)	306 (372)	0.406
Ketamine consumption (mg) at 48 h, mean (SD)	323 (394)	266 (287)	455 (588)	0.481
Ketamine consumption (mg) at 72 h, mean (SD)	374 (455)	310 (354)	521 (652)	0.483

**Table 4:** Correlation between STAI-6 and NRS-11, complication rates, length of stay, and readmission rates

Parameter	Any complications	Readmission	NRS-11 (24 h)	Length of Stay
N	319	315	319	319
STAI-6 (24 h)*	1.00 (0.98, 1.01)	1.00 (0.98, 1.03)	0.07 (0.05, 0.09)	0.04 (0.02, 0.06)

*Odds ratio (95%Confidence Interval)

**Table 5:** Correlation between STAI-6 and NRS-11, complication rates, length of stay, and readmission rates

	[ALL]	Music	Silence	p-value
	*N* = *317*	*N* = *135*	*N* = *182*	
Preoperative NRS-11, mean (SD)	2.92 (2.76)	2.72 (2.43)	3.07 (2.99)	0.247
NRS-11 at 24 ± 3 h, mean (SD)	5.22 (2.54)	4.99 (2.46)	5.39 (2.60)	0.162
Preoperative STAI-6, mean (SD)	40.2 (14.2)	40.6 (14.5)	40.0 (13.9)	0.691
STAI-6 at 24 ± 3 h, mean (SD)	35.4 (14.4)	31.1 (11.9)	38.7 (15.3)	< 0.001
Length of stay (days), mean (SD)	5.60 (2.75)	5.27 (2.36)	5.84 (3.00)	0.063
Readmission, n (%)	26 (8.31%)	14 (10.4%)	12 (6.74%)	0.344
Any complication, n (%)	109 (34.4%)	43 (31.9%)	66 (36.3%)	0.485

*Subgroup analysis:* There were 100 patients randomized to music and 97 randomized to silence without preoperative chronic opioid use or diagnosis of anxiety. All subgroup analyses are summarized in Supplement 2.

## Discussion

In this double-blind, placebo-controlled randomized trial, intraoperative music did not reduce patient-reported pain scores on the first postoperative day in patients undergoing open AWR. Furthermore, there was no apparent benefit to intraoperative music regarding anxiety, postoperative opioid consumption, or intraoperative sedative requirement. Although patients who believed they listened to music intraoperatively demonstrated lower anxiety scores at 24 h postoperatively than those who believed they listened to silence, which was itself associated with lower pain scores and shorter length of stay.

Abdominal wall reconstruction is painful. Contemporary literature has examined several modalities for pain control including TAP blocks, epidurals, and non-narcotic medications. In a randomized trial of TAP blocks in patients undergoing open AWR, Fafaj et al. demonstrated high opioid requirements within 72 h postoperatively, without reduction between treatment and placebo arms [1]. A retrospective review of epidural analgesia in AWR surprisingly found worse patient-reported pain scores compared to standard Enhanced Recovery After Surgery protocol and increased complication rates (26% vs 21%, p < 0.05) [21]. Non-narcotic medications such as gabapentin or non-steroidal anti-inflammatory drugs may be used as adjunctive analgesics, but are not tolerated or appropriate for use in all populations [22, 23]. In contrast to alternative adjuncts, music is sustainable, inexpensive, accessible, and presents minimal risks. Additionally, there is evidence that people form implicit memory under general anesthesia, supporting the idea that intraoperative music may be beneficial [13, 24, 25].

Numerous RCTs and meta-analyses have elucidated benefits of perioperative music including reduced pain, anxiety, and length of stay [4–8, 26]. Music reduces pain perception during conscious procedures [27–30]. In a double-blinded trial of patients undergoing hysterectomy, intraoperative music was associated with lower patient-perceived pain and opioid requirements [26]. The same authors found reduced patient-reported pain and opioid requirements with music administered preoperatively, intraoperatively, and postoperatively for patients undergoing inguinal and ventral hernia repairs at ambulatory surgery centers [7, 8, 10]. While these trials provide some evidence for pain reduction with perioperative music, readers may criticize the lack of blinding associated with pre- and postoperative music. Furthermore, in a systematic review and meta-analysis of RCTs, Fu et al. found that only 18% of trial included music solely intraoperatively and many of those studied surgeries performed under regional or spinal anesthesia, leading the authors to acknowledge a high risk for performance and detection biases based on the lack of blinding [5]. The only way to blind both patients and assessors to therefore mitigate these potential biases is to administer the intervention intraoperatively while the patient is sedated under general anesthesia. Biases aside, it is also possible that for less painful operations, isolated intraoperative music might be a helpful adjunct, but may be less efficacious for major surgeries.

While our trial did not find benefits in any outcome measures, the methodology employed ensured lower bias than much of the preceding literature. One of the greatest strengths of this trial is the blinding of patients, operating room personnel, and assessors. The music was administered only during general anesthesia, all patients wore headphones intraoperatively, and the only person in the room aware of allocation was a study coordinator uninvolved in assessments. This minimized the possibility for unblinding and bias. While many RCTs have demonstrated modest reduction in postoperative opioid consumption [31–33], meta-analysis of well-blinded trials did not show a difference in opioid consumption in [5, 26, 34, 35]. Szmuk et al. reported a similar double-blind, RCT in which patients received music or silence through headphones during general anesthesia and analogously to our findings, there was no difference in intraoperative sedation requirements as measured by end-tidal concentration of sevoflurane necessary to maintain bispectral index near 50 during laparoscopic surgery [36]. Additionally, objective clinical outcomes such as length of stay have not demonstrated consistent reduction with perioperative music [33, 37, 38]. Taken in context with the existing literature, these results suggest that in intraoperative music studies with appropriate blinding the true benefits of music in this surgical population may be negligible.

While the findings of our trial certainly question the benefits of intraoperative music in open AWR, it is important to note that this population tested the limits of music. Nilsson et al. has published prolifically on the topic of music including multiple blinded, RCTs showing positive effects of intraoperative music during general anesthesia, however the populations represented mostly ambulatory surgeries and excluded patients with psychiatric disorders or substance use history [8, 26]. Nearly one-third of our patients reported an anxiety disorder, 15.6% were classified as chronic opioid users, most patients were classified as ASA Class III, and mean hernia width was 14.5 cm, highlighting both the medical and surgical complexity of our population. Furthermore, the prevalence of post-traumatic stress disorder in patients with incisional hernias is approximately 32.1%, which perhaps contributed to perioperative anxiety for these patients [39]. To address this divergence from existing literature, additional subgroup analyses of pain, anxiety, and opioid consumption were performed that excluded patients with chronic opioid use or preoperative anxiety. Those analyses similarly demonstrated no benefit to music with regards to patient-reported pain and anxiety or opioid consumption postoperatively. However, as an exploratory subgroup analysis these results cannot dismiss the potential therapeutic effect of music in patients without chronic pain or anxiety.

There are limitations to this trial. The patients represented constitute a particularly medically and surgically complex group, limiting the generalizability of these results. Compared to many previous trials with music, this surgical population underwent extensive, painful operations and had high baseline rates of chronic pain, opioid use, and anxiety. Though all patients underwent retromuscular dissection, inclusion criteria allowed for multiple hernia types including midline, parastomal, and flank, which may introduce heterogeneity in expected levels of postoperative pain, though allocations were even between groups. Patients who ultimately did not undergo retromuscular abdominal wall reconstruction were considered dropouts and excluded from postoperative data collection given the expectations for lower postoperative pain, but this could be seen as a limitation. The choice to restrict the intervention to the intraoperative period allowed for blinding to minimize performance and detection bias but also precludes the interpretation of music’s efficacy as an analgesic adjunct for conscious patients. There may be positive effects to music therapy preoperatively or postoperatively, but this was beyond the scope of the present trial. Silence may be a non-ideal comparison, considering standard of care would be the ambient noise of operating rooms. Additionally, sedation protocols could not practically be standardized for the purpose of this study. For example, benzodiazepines, which may impair implicit memory formation, were allowed at the discretion of the anesthesiologist [40].

## Conclusion

Intraoperative music does not appear to confer benefits regarding patient-reported pain scores following open AWR with mesh. Further work should focus on low-cost, low-risk, accessible and sustainable pain management strategies with a goal of reducing postoperative patient-reported pain and opioid usage.

## Trial registration

ClinicalTrials.gov Identifier: NCT05374096, https://clinicaltrials.gov/study/NCT05374096

## Supplementary Information

Below is the link to the electronic supplementary material.

Supplementary file1 (DOCX 19 KB)

Supplementary file2 (EPS 15 KB)

Supplementary file3 (DOCX 173 KB)

## Data Availability

We do not plan to make data publicly available.

## Abbreviations

AWR: Abdominal wall reconstruction
ACHQC: Abdominal Core Health Quality Collaborative
REDCap: Research Electronic Data Capture
MME: Morphine milligram equivalents
ASA: American Society of Anesthesiologists
NRS-11: Numeric Rating Scale
STAI-6: State-Trait Anxiety Inventory
SSI: Surgical site infection
SSO: Surgical site occurrence
SSOPI: Surgical site occurrence requiring procedural intervention
