# Supplementary material for: Does selective intraoperative music reduce pain following abdominal wall reconstruction? A double-blind randomized controlled trial
Source: Hernia. 2024 Jun 18;28(5):1831–41. doi: 10.1007/s10029-024-03092-y (PMC11450090; doi:10.1007/s10029-024-03092-y)
Supplement: Supplementary file 1 — Supplementary file1 (DOCX 19 KB) [file 10029_2024_3092_MOESM1_ESM.docx]

**Supplemental Tables:**

**Table 1: Postoperative course**

|  | **[ALL]** | **Music** | **Silence** | **p-value** |
| --- | --- | --- | --- | --- |
|  | ***N=321*** | ***N=164*** | ***N=157*** |  |
| Readmission, n (%) | 28 (8.83%) | 16 (9.88%) | 12 (7.74%) | 0.637 |
| Reoperation, n (%) | 5 (1.58%) | 2 (1.23%) | 3 (1.94%) | 0.679 |
| Length of stay (days), mean (SD) | 5.60 (2.75) | 5.43 (2.52) | 5.77 (2.96) | 0.273 |
| Postoperative SSI, n (%) | 33 (10.4%) | 14 (8.64%) | 19 (12.3%) | 0.384 |
| Postoperative SSOPI, n (%) | 32 (10.1%) | 16 (9.88%) | 16 (10.3%) | 1.000 |
| Postoperative SSO, n (%) | 46 (14.5%) | 21 (13.0%) | 25 (16.1%) | 0.522 |
| Readmission for pain, n (%) | 16 (5.05%) | 10 (6.17%) | 6 (3.87%) | 0.497 |
| Deep vein thrombosis, n (%) | 4 (1.26%) | 1 (0.62%) | 3 (1.94%) | 0.362 |
| Sepsis, n (%) | 1 (0.32%) | 1 (0.62%) | 0 (0.00%) | 1.000 |
| Myocardial infarction, n (%) | 1 (0.32%) | 1 (0.62%) | 0 (0.00%) | 1.000 |
| Urinary tract infection, n (%) | 10 (3.15%) | 5 (3.09%) | 5 (3.23%) | 1.000 |
| Acute renal failure, n (%) | 5 (1.58%) | 3 (1.85%) | 2 (1.29%) | 1.000 |
| Pneumonia, n (%) | 13 (4.10%) | 8 (4.94%) | 5 (3.23%) | 0.627 |
| Endotracheal intubation, n (%) | 2 (0.63%) | 1 (0.62%) | 1 (0.65%) | 1.000 |

**Table 2. Subgroup analysis: NRS-11 in the first three postoperative days**

| Parameter | Coefficient | SE | 95% CI | t(581) | p |
| --- | --- | --- | --- | --- | --- |
| (Intercept) | 3.84 | 0.24 | (3.36, 4.32) | 15.72 | < .001 |
| Randomization treatment (Silence vs music) | 0.22 | 0.27 | (-0.32, 0.75) | 0.79 | 0.428 |
| Preoperative NRS-11 | 0.38 | 0.05 | (0.27, 0.49) | 6.94 | < .001 |
| NRS-11 at 48±3 hours | -0.72 | 0.16 | (-1.04, -0.40) | -4.38 | < .001 |
| NRS-11 at 72±3 hours | -1.37 | 0.16 | (-1.69, -1.05) | -8.33 | < .001 |

**Table 3. Subgroup analysis: STAI-6 in the first three postoperative days**

| Parameter | Coefficient | SE | 95% CI | t(579) | p |
| --- | --- | --- | --- | --- | --- |
| (Intercept) | 22.06 | 2.47 | (17.20, 26.92) | 8.91 | < .001 |
| Randomization treatment (Silence vs music) | -1.15 | 1.48 | (-4.06, 1.75) | -0.78 | 0.437 |
| STAI-6 at 48±3 hours | -0.85 | 1.00 | (-2.82, 1.12) | -0.85 | 0.396 |
| STAI-6 at 72±3 hours | -1.61 | 1.00 | (-3.58, 0.35) | -1.61 | 0.108 |
| Preoperative STAI-6 | 0.33 | 0.06 | (0.22, 0.44) | 5.99 | < .001 |

**Table 4. Subgroup analysis: Opioid consumption (MME) in the first three postoperative days**

|  | **[ALL]** | **Music** | **Silence** | **p-value** |
| --- | --- | --- | --- | --- |
|  | ***N=197*** | ***N=100*** | ***N=97*** |  |
| Intraoperative opioid consumption (MME), mean (SD) | 75.1 (52.8) | 79.0 (70.5) | 71.2 (23.5) | 0.299 |
| Opioid consumption (MME) at 24±3 hours, mean (SD) | 163 (348) | 198 (478) | 127 (97.4) | 0.147 |
| Opioid consumption (MME) at 48±3 hours, mean (SD) | 248 (516) | 299 (706) | 196 (154) | 0.156 |
| Opioid consumption (MME) at 72±3 hours, mean (SD) | 395 (1457) | 355 (751) | 436 (1936) | 0.700 |

Supplemental 2 Figure Legend: Subgroup analysis of pain (NRS-11) and anxiety (STAI-6) scores over the first 72 hours postoperatively
