# Supplementary material for: Does selective intraoperative music reduce pain following abdominal wall reconstruction? A double-blind randomized controlled trial
Source: Hernia. 2024 Jun 18;28(5):1831–41. doi: 10.1007/s10029-024-03092-y (PMC11450090; doi:10.1007/s10029-024-03092-y)
Supplement: Supplementary file 3 — Supplementary file3 (DOCX 173 KB) [file 10029_2024_3092_MOESM3_ESM.docx]

#
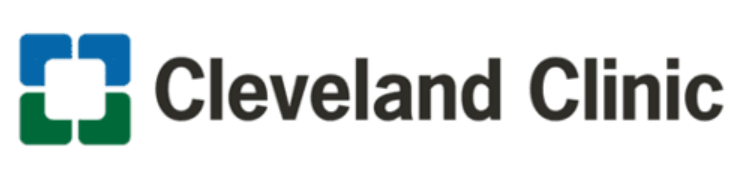


Center for Abdominal Core Health

**The effect of intraoperative music on pain in patients undergoing ventral hernia repair with mesh: a double-blind randomized controlled trial**

Version 5, 1/15/2023

Primary Investigator:

Ajita Prabhu, MD

Co Investigators:

Sara Maskal, MD
 Ryan Ellis, MD

Corey Gentle, MD

George Saieed

Katie Montelione, MD

Benjamin Miller, MD

Kaela Parnell, MD

Clayton Petro, MD

Luke Beffa, MD

David Krpata, MD

Steven Rosenblatt, MD

Michael Rosen, MD

Digestive Diseases and Surgery Institute, Cleveland Clinic Center for Abdominal Core Health, Department of General Surgery, Cleveland Clinic, Main Campus, 9500 Euclid Ave — A100, Cleveland, OH 44195, Tel: 216 444 4790, email: [prabhua@ccf.org](https://clevelandclinic-my.sharepoint.com/personal/gentlec_ccf_org/Documents/Music%20RCT/prabhua@ccf.org)

## Background

Open abdominal wall reconstruction is often performed for large or recurrent ventral hernias, which involves a midline laparotomy, different levels of myofascial release, and considerable dissection to separate the components of the abdominal wall which can cause significant pain. Multiple pain management strategies have been applied to this particular patient population, with little success. Epidural analgesia is a widely used method to control pain from thoracic to gynecologic surgery,^1^ and has even been shown to decrease length of stay in orthopedic^2^ and colorectal^3^ surgeries. However, not only did patients have no less pain after complex ventral hernia repair when given epidural analgesia, but length of stay was in fact longer in this group.^4^ Another non-opioid pain management strategy called the transversus abdominis plane (TAP) block delivers local anesthetic around the nerves in an intra-muscular plane to achieve pain control in the abdominal wall. A randomized controlled trial of liposomal and non-liposomal bupivacaine TAP blocks in abdominal wall reconstruction found no differences in post-operative pain or opioid use between the placebo group and the TAP block groups.^5^

Due to the lack of compelling evidence for these non-opioid based regimens, our current strategy involves pain control using patient-controlled analgesia (PCA), in which patients have on-demand access to intravenous opioid medications for immediate post operative pain control. This is problematic given that the treatment of postoperative pain with narcotic pain medication is well known to have contributed to the opioid epidemic in the United States, leading to a high rate of opioid related drug overdose deaths.^6^ Furthermore, surgical patients also often suffer from preoperative anxiety which further increases the level of postoperative pain, potentially further driving use of narcotic pain medication. ^7^

Given these issues, clinicians must continue to explore non-opioid pain management strategies for surgical patients. Meta analyses of randomized controlled trials have shown that perioperative music significantly reduced both pain and anxiety in adult surgical patients.^8,9^ Perioperative music also has a significant beneficial effect on postoperative opioid requirement and can attenuate the physiological stress response to surgery with less need for sedatives during general anesthesia.^10,11^ Several studies show that auditory sensory information is still processed during general anesthesia and recent meta-analyses have reported beneficial effects of music even if solely played during general anesthesia.^8–10^ Still, the noted meta-analyses are limited by heterogeneity of surgical procedures, diverse methods of anesthesia, and variability in the time frame in which the interventions were offered. While existing literature suggests that music may attenuate anxiety and pain in surgical patients, no specific literature has explored the effect of intraoperative music on pain and anxiety on patients undergoing ventral hernia repair. The aim of this study is to determine whether intraoperative music decreases post operative pain and anxiety after open ventral hernia repair.

## Specific Aims

Previous work has shown that intraoperative music can mitigate postoperative pain and anxiety in patients undergoing surgery. Presumably, intraoperative music based on patient preference during ventral hernia repair would result in lower postoperative pain and anxiety scores and would therefore also lower the amount of postoperative opioid pain medication taken by patients who have undergone ventral hernia repair.

### Aim of this study:

Demonstrate that intraoperative music (defined as after induction of anesthesia) decreases postoperative pain and anxiety scores in patients undergoing ventral hernia repair.

**Primary hypothesis**. Pain scores as measured by NRS-11 (0-10 verbal response scale)^12^ the first day after surgery are lower in patients with intraoperative music than with routine care, with 1.8 points being considered a clinically meaningful difference.

**Primary Outcome.** Pain Scores (NRS-11) measured once pre-operatively and at 24 ± 3 hrs after surgery is complete. We will then use the difference between these two scores to account for baseline pain.

**Secondary hypothesis.** Patients who receive intraoperative music have lower anxiety scores as measured by the State Trait Anxiety Inventory (STAI-6)^13^ on the first post operative day, with a 10-point decrease in scores being considered clinically meaningful.^14^

**Secondary Outcome.** Anxiety level (using STAI-6 Survey) taken at 24 ± 3 hrs after surgery is complete.

We will also consider several exploratory hypotheses.

**Exploratory hypothesis 1**. NRS-11 pain scores are lower in the music group than the control group over the first 3 postoperative days (to be recorded at 24 ± 3 hours, 48 ± 3 hours and 72 ± 3 hours).

**Exploratory hypothesis 2** The cumulative pain recorded in the first 24 hours will be lower in the music group than the control measured using area under the pain-time curve.

**Exploratory hypothesis 3**. STAI 6 scores are lower in the music group than the control group over the first 3 postoperative days (survey to be completed at 24 ± 3 hours, 48 ± 3 hours and 72 ± 3 hours).

**Exploratory hypothesis 4**. Patients in the music group use less opioid pain medication during the first 72 hours after surgery measured in morphine milligram equivalents.

**Exploratory hypothesis 5** Patients in the music group require less sedative medication use during surgery.

**Exploratory hypothesis 6** STAI 6 scores will be positively correlated with postoperative NRS-11 pain scores, complication rates, length of stay, and readmission rates.

**Exploratory hypothesis 7** Patients who believe that they were listening to music will show lower STAI 6 scores, NRS-11 pain scores, rate of complications, length of stay, and rates of readmission.

## Methods

The study will be conducted with IRB approval and written informed consent. The trial will be registered at ClinicalTrials.gov before the first patient is enrolled.

### Data Sources

This study will be performed utilizing existing data collection efforts from the Abdominal Core Health Quality Collaborative (ACHQC). Cleveland Clinic is a participant of the ACHQC. The ACHQC is an organization dedicated to quality improvement in hernia and abdominal core health and already receives protected health information from this institution for routine healthcare operations under the terms of a business associate agreement. This pre-existing data collection effort will be utilized for the research study. Additional data elements, as specified in this protocol, may be combined with the ACHQC data to create a final research dataset.

The data flow for this study is as outlined in figure:


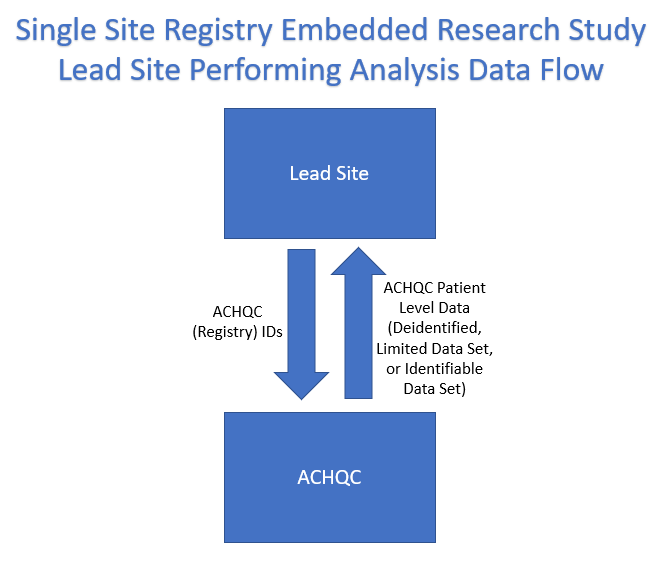


### Subject selection

We will enroll patients having open retromuscular ventral hernia repair with mesh. Patients who chronically use opioids will *not* be excluded (definition of chronic opioid use below). Both men and women will be recruited, and we will encourage under-represented minorities to participate. See Sample Size Considerations regarding expected enrollment.

#### Inclusion criteria

1. Adults having open retromuscular ventral hernia repair with mesh, with or without myofascial release for a hernia width ≤ 20 cm
2. Adults having open flank hernia repair that requires a myofascial release with mesh
3. Adults having parastomal hernia repair with mesh

#### Exclusion criteria

1. Primary language other than English, or lack of English language fluency
2. Hearing impairment, with or without use of hearing aids
3. Neurologic condition that, in the opinion of the investigators, may preclude accurate assessment of postoperative pain and anxiety
4. Patients who will remain intubated after surgery

### Protocol

During their pre-operative visit after consent is obtained, patients will be asked to choose from a list of pre-selected music genres which one they would prefer to listen to during surgery. This will be recorded and prepared for every patient prior to the day of surgery.

On the day of surgery, general anesthesia will be induced as preferred by the attending anesthesiologist. Vasopressors, antihypertensives, and drugs to control heart rate may be given as clinically indicated. Headphones will be placed after induction of anesthesia and prior to incision for surgery.  After the headphones are placed, randomization will occur.

**Randomization**

Patients will be randomized 1:1, stratified for pre-operative chronic opioid use (2 levels, opioid use and no opioid use), with random-sized blocking to patient-selected music administered through headphones or routine care with headphones and no music. The randomization table will be prepared by trial statisticians, and allocation will be concealed until shortly before anesthetic induction with a web-based randomization system.

1. **Routine care with headphones.**  After induction of anesthesia and prior to incision for surgery, headphones will be placed. Music will not be started. Headphones will be removed at the beginning of emergence from anesthesia.
2. **Music administration.** After induction of anesthesia and prior to incision for surgery, headphones will be placed with the patient-selected music started immediately after randomization. Music administration will continue until the beginning of emergence from anesthesia, at which point the music will be stopped and headphones will be removed.

As headphones will be placed for both treatment and control groups by a member of the research team who is not blinded, and the music device will be controlled by this individual. Anesthesiologists and operating surgeons will both be blinded to randomization and have no interaction with the headphones or music playing device. For all patients, attending anesthesiologist will use clinical judgment to determine how much narcotic pain medication should be given, and when. Clinical judgment will be according to their standard practice and may include interpretation of blood pressure, heart rate, diaphoresis, tearing, and pupil size. All patients will be given an intra-operative transversus abdominis plane (TAP) block by the operative surgeon.

One anticipated scenario in which patients will be randomized prior to assessment of all exclusion criteria. This will occur when the operating surgeon/anesthesiologist deem it necessary for a patient to be transferred from the operating room to the intensive care unit intubated for the beginning of post-operative recovery. If a patient is randomized, but no surgeon on the protocol scrubs in to perform either initial dissection or closure, then they will be considered a screen failure and will not be included in the final analysis. Another scenario that will be considered a screen failure after randomization is if the patient has an unexpected intra-abdominal or soft tissue finding that requires a significant change in operative plan and precludes hernia repair with mesh. Additionally, we intend for all patients in this study to undergo hernia repair with no trans fascial fixation of mesh, as this causes significant additional pain, but recognize that some patients may undergo fixation based on surgeon assessment of intra-operative findings. Both scenarios are more likely when the hernia width is larger, and we have tried to minimize these occurrences by limiting inclusion criteria to hernias ≤ 20 cm in width. When a patient leaves the operating room intubated for clinical safety, the patient will be considered an exclusion after randomization and not be included in any final analysis. When the operating surgeon deems trans fascial fixation of the mesh necessary during the operation, this will be recorded. The patient will be included in all subsequent analyses based on the group to which they were originally randomized.

The same individual who placed the headphones at the start of the case will return to remove the headphones and stop the music in the treatment group at the time of emergence. A this individual is un-blinded, they will not be involved in any post-operative data collection.

Postoperative care in the Post Anesthesia Care Unit (PACU) will be as per standard care per PACU RNs and anesthesia attendings.

### Measurements

**Pre-Operative:** Demographic and morphometric characteristics will be recorded, including sex, age, race, height, weight, opioid use in the last month (estimated morphine milligram equivalents), opioid use in the last year, baseline pain using NRS-11, and baseline anxiety (both STAI-6 measure and based on if patient self-reports a prior diagnosis of any anxiety disorder). Name is necessary in the REDCap in order to track when the subject is planned for surgery and to verify identity prior to randomization, which will typically occur in the operating room and will be difficult using MRN alone. MRN is necessary in order to track and access the electronic medical record to access multiple data points including pain scores in the first 24 hours, pain medication use, sedation, and postoperative outcomes as listed below. These identifiers will only be stored on Cleveland Clinic password protected computers. Chronic opioid use will be defined as daily or near daily use of opioids for ≥ 90 days in the past year.^15^ Patient preferred music genre will also be recorded at this time.

**Intra-Operative:** Surgical care of the patient will be carried out as per routine. Hernia width will be recorded, as well as whether or not myofascial release and mesh fixation are performed. Sedative use during the case will be recorded, including intravenous and inhaled anesthetics, benzodiazepines, and opioids. We will record the start time of music (which will fall between induction and incision), the time at which music is discontinued (the end of anesthesia), along with the end of surgery (last stitch), and extubation. Discontinuation of inhaled anesthetic will be considered the end of anesthesia and the beginning of recovery.

**Post-Operative:** All postoperative care and evaluations will be fully blinded to randomization, thus limiting measurement bias. In the PACU, patients will be asked to rate their pain at rest on a 0-10 verbal response scale. Somnolent patients will be given a score of “S” for queries to which they are unable to respond. The STAI-6 will be administered first on post operative day 1. Additionally, at the end of the case the anesthesia team will be asked to predict whether or not the patient was listening to any music based on their blinded assessment of the patient during surgery.

All subsequent management will be per clinical routine, and pain scores will be recorded as per RN standard on the floor. Pain management post operatively by the surgical team will follow the surgeon’s standard of practice. Study personnel will ensure a 24 hour ± 3-hour pain score is recorded for every patient. The STAI-6 will be administered by the postoperative study personnel daily while in the hospital until and including Postoperative Day #3. All post operative opioid use will be recorded in morphine milligram equivalents, and any non-opioid pain control adjunct medications will also be recorded.

Data will retrospectively be collected from the 30 (+/- 15) day postoperative visit which is standard of care. This includes: total length of stay, ileus, bowel obstruction, pain requiring intervention, DVT/PE, stroke, sepsis, septic shock, MI, cardiac arrest, urinary retention, UTI, renal insufficiency, acute renal failure, pneumonia, respiratory failure, ventilator >48 hours, surgical site infection, surgical site occurrence, surgical site occurrence requiring procedural intervention, reoperation, and readmission.

### Data Analysis

Randomized groups will be assessed for balance on baseline characteristics using the standardized differences, defined as the difference in means or proportions divided by the pooled standard deviation. An absolute standardized differences > 0.10 will be considered as imbalanced. We will use intent-to-treat such that all randomized patients receiving any of the study intervention (either one, even if the incorrect treatment was applied) will be included in the primary analyses.

**Primary outcome**. We will assess the treatment effect of music versus routine care on pain score at 24±3 hours after completion of the procedure using linear regression with pre-specified covariates (chronic opioid use, hernia width, operative time, myofascial release, pre-operative anxiety disorder diagnosis, and pre-operative STAI-6 anxiety score). For patients who have a pre-operative pain score > 0, we will calculate the difference in pre- and post-operative scores to account for baseline pain.

*Sensitivity analysis.*  As a sensitivity analysis we will assess the treatment effect on pain score using a proportional odds model estimating the proportional odds ratio for patients having a better outcome on NOL than standard care, still adjusting for the pre-specified variable.

**Secondary outcome.** We will assess the treatment effect of music versus routine care on the STAI 6 anxiety questionnaire at 24±3 hrs post surgery using linear regression with pre-specified covariates (chronic opioid use, hernia width, operative time, myofascial release, pre-operative anxiety disorder diagnosis, and pre-operative STAI-6 anxiety score).

**Exploratory outcome 1.** We will assess the treatment effect of intraoperative music on the first 3 postoperative days of music on pain scores NRS-11 using mixed effect linear regression model with treatment group and pre-specified covariates as fixed effect and the repeated measurements as random effect.

**Exploratory Outcome 2.** We will assess the treatment effect of intraoperative music on the area under the curve of the pain time curve by plotting NRS-11 pain scores in time during the first 24 hours post operatively. Sum Pain Intensity Differences Area Calculation for all measurements of pain score during first 24 hours would be used. Simple two sample t-test would be used in this comparison.

**Exploratory Outcome 3.** We will assess the treatment effect of intraoperative music on the STAI-6 scores over the first 3 postoperative days using mixed effect linear regression model with treatment group and pre-specified covariates as fixed effect and the repeated measurements as random effect.

**Exploratory Outcome 4.** We will assess the treatment effect of intraoperative music on consumption of opioids (converted to morphine milligram equivalents [MME]) during the postoperative period defined as the first 72 hours after surgery.

**Exploratory Outcome 5.** We will assess the treatment effect of intraoperative music on intraoperative sedative use defined from induction to arrival to PACU.

**Exploratory Outcome 6.** Logistic regression models will be applied with dependent variable complications, readmission and independent variable STAI 6 anxiety scores. Linear regression models will be used with dependent variable postoperative pain scores, length of stay and independent variable STAI 6 anxiety scores. Mixed model will also be considered given some outcomes are repeatedly measured.

**Exploratory Outcome 7.** Patients will be separated into two groups according to the question “do you think you were listening to music or silence?” and compared using STAI 6 scores, NRS-11 pain scores, rates of complications, length of stay and rates of readmission.

**Sample size justification**.

1. **Patients for power calculation:** A sample of patients who underwent retromuscular ventral hernia repair without fixation were used to calculate the sample size for this study. In the below sample size calculations, we used pain at 24±12 hours post operatively on these patients to help estimate the standard deviation of the primary outcome (pain score).
2. **Required Sample Size.** The primary outcome parameter is used in the power calculation. Difference in the NRS-11 pain score between the two groups were compared using Student’s t-test with equal variance. Calculations were done using SAS software (version 9.4, Cary, NC).

In the above sample of 100 patients, the average score at 24±12 hours is 4.52 with standard deviation 2.47. Using this mean and standard deviation, we have chosen our sample size at 320 total patients with 160 in each arm to detect a 20% reduction in pain score with a 90% power.

1. **Interim analyses.** Given that the risk of harm of the intervention is minimal, we will not be conduction interim analyses in this study.

## Human Subjects Protection

Typical pain scores during the initial phase of post-anesthetic recovery at the Cleveland Clinic Main Campus are 4.7/10, indicating moderate pain control. The only aspect of our protocol that is non-standard is administration of music vs standard care. Pain scores could hardly be worse with music administration, and in all cases, clinical judgement will prevail. Standard vital signs monitoring will continue to be used per current routine care. Our experienced anesthesia attending will never give patients clearly inadequate or excessive doses of pain medications.

The following Adverse Events (AE) will be collected:

- All AEs with a surgery-related cause
- All Serious Adverse Events (SAE) (including sepsis events or related to opioid therapy)

Pre-planned interventions or occurrence of endpoints, including deviations in vital signs, specified in the CIP are not considered AEs, if not defined otherwise

Table 1. Definition of Adverse Events

| **Term** | **Abbreviation** | **ISO Definition** |
| --- | --- | --- |
| Adverse Event | AE | Any untoward medical occurrence, unintended disease or injury, or untoward clinical signs (including abnormal laboratory findings) in subjects, users or other persons, whether or not related to the investigational medical device.  *NOTE 1:* This definition includes events related to the investigational medical device or the comparator.  *NOTE 2*: This definition includes events related to the procedures involved.  *NOTE 3:* For users or other persons, this definition is restricted to events related to investigational medical devices. |

####

#### Reporting procedures

AE information will be collected throughout the study and reported to the IRB. It is the responsibility of the investigator to identify the occurrence of adverse events to ensure that the information is accurately documented in the medical record and on the eCRFs.

AE documentation will include the following information at a minimum:

- Date of event
- Time of the event
- Diagnosis and description
- Actions taken / treatment (including vital signs, and date and time of rescue related actions when applicable)
- Assessment of seriousness
- Outcome or resolution and date of the resolution

## Supplemental Material

1. **NRS-11 pain scale**
2. **STAI-6 anxiety tool**

## Funding

This study project is an investigator-initiated project and has no specific funding.

## References

1. Pöpping DM, Zahn PK, van Aken HK, Dasch B, Boche R, Pogatzki-Zahn EM. Effectiveness and safety of postoperative pain management: a survey of 18 925 consecutive patients between 1998 and 2006 (2nd revision): a database analysis of prospectively raised data. *British Journal of Anaesthesia*. 2008;101(6). doi:10.1093/bja/aen300

2. Neuman MD, Rosenbaum PR, Ludwig JM, Zubizarreta JR, Silber JH. Anesthesia Technique, Mortality, and Length of Stay After Hip Fracture Surgery. *JAMA*. 2014;311(24). doi:10.1001/jama.2014.6499

3. Senagore AJ, Whalley D, Delaney CP, Mekhail N, Duepree HJ, Fazio VW. Epidural anesthesia-analgesia shortens length of stay after laparoscopic segmental colectomy for benign pathology. *Surgery*. 2001;129(6). doi:10.1067/msy.2001.114648

4. Prabhu AS, Krpata DM, Perez A, et al. Is It Time to Reconsider Postoperative Epidural Analgesia in Patients Undergoing Elective Ventral Hernia Repair? *Annals of Surgery*. 2018;267(5). doi:10.1097/SLA.0000000000002214

5. Fafaj A, Krpata DM, Petro CC, et al. The Efficacy of Liposomal Bupivacaine On Postoperative Pain Following Abdominal Wall Reconstruction. *Annals of Surgery*. Published online December 2, 2020. doi:10.1097/SLA.0000000000004424

6. Scholl L, Seth P, Kariisa M, Wilson N, Baldwin G. Drug and Opioid-Involved Overdose Deaths — United States, 2013–2017. *MMWR Morbidity and Mortality Weekly Report*. 2018;67(5152). doi:10.15585/mmwr.mm675152e1

7. Ip HYV, Abrishami A, Peng PWH, Wong J, Chung F. Predictors of Postoperative Pain and Analgesic Consumption. *Anesthesiology*. 2009;111(3):657-677. doi:10.1097/aln.0b013e3181aae87a

8. Kühlmann AYR, de Rooij A, Kroese LF, van Dijk M, Hunink MGM, Jeekel J. Meta-analysis evaluating music interventions for anxiety and pain in surgery. *British Journal of Surgery*. 2018;105(7):773-783. doi:10.1002/bjs.10853

9. Hole J, Hirsch M, Ball E, Meads C. Music as an aid for postoperative recovery in adults: a systematic review and meta-analysis. *The Lancet*. 2015;386(10004). doi:10.1016/S0140-6736(15)60169-6

10. Fu VX, Oomens P, Klimek M, Verhofstad MHJ, Jeekel J. The Effect of Perioperative Music on Medication Requirement and Hospital Length of Stay. *Annals of Surgery*. 2019;XX(Xx):1. doi:10.1097/sla.0000000000003506

11. Fu VX, Oomens P, Sneiders D, et al. The Effect of Perioperative Music on the Stress Response to Surgery: A Meta-analysis. *Journal of Surgical Research*. 2019;244:444-455. doi:10.1016/j.jss.2019.06.052

12. Dworkin RH, Turk DC, Farrar JT, et al. Core outcome measures for chronic pain clinical trials: IMMPACT recommendations. *Pain*. 2005;113(1). doi:10.1016/j.pain.2004.09.012

13. Marteau TM, Bekker H. The development of a six‐item short‐form of the state scale of the Spielberger State—Trait Anxiety Inventory (STAI). *British Journal of Clinical Psychology*. 1992;31(3). doi:10.1111/j.2044-8260.1992.tb00997.x

14. Corsaletti BF, Proenca MDGL, Bisca GKW, Leite JC, Bellinetti LM, Pitta F. Minimal important difference for anxiety and depression surveys after intervention to increase daily physical activity in smokers. *Fisioter Pesqui*. 2014;21(4).

15. Korff M von, Saunders K, Thomas Ray G, et al. De Facto Long-term Opioid Therapy for Noncancer Pain. *The Clinical Journal of Pain*. 2008;24(6). doi:10.1097/AJP.0b013e318169d03b
